# Activation of the C-Type Lectin MGL by Terminal GalNAc Ligands Reduces the Glycolytic Activity of Human Dendritic Cells

**DOI:** 10.3389/fimmu.2020.00305

**Published:** 2020-02-25

**Authors:** Anouk Zaal, R. J. Eveline Li, Joyce Lübbers, Sven C. M. Bruijns, Hakan Kalay, Yvette van Kooyk, Sandra J. van Vliet

**Affiliations:** Department of Molecular Cell Biology and Immunology, Cancer Center Amsterdam, Amsterdam Infection and Immunity Institute, Amsterdam UMC, Vrije Universiteit Amsterdam, Amsterdam, Netherlands

**Keywords:** macrophage galactose-type lectin, antigen presenting cells, tumor-associated glycans, metabolism, glycolysis, oxidative phosphorylation, TCA cycle, glycodendrimers

## Abstract

Many tumors display alterations in the biosynthetic pathways of glycosylation, resulting in increased expression of specific tumor-associated glycan structures. Expression of these altered glycan structures is associated with metastasis and poor prognosis. Antigen presenting cells can recognize tumor-associated glycan structures, including the truncated *O*-glycan Tn antigen, via specific glycan receptors. Tn antigen-mediated activation of the C-type lectin MGL on dendritic cells induces regulatory T cells via the enhanced secretion of IL-10. Although these findings indicate that MGL engagement by glycan ligands can modulate immune responses, the impact of MGL ligation on dendritic cells is still not completely understood. Therefore, we employed RNA sequencing, GO term enrichment and pathway analysis on human monocyte-derived dendritic cells stimulated with two different MGL glycan ligands. Our analyses revealed a reduced expression of genes coding for key enzymes involved in the glycolysis pathway, TCA cycle, and oxidative phosphorylation. In concordance with this, extracellular flux analysis confirmed the decrease in glycolytic activity upon MGL triggering in human dendritic cells. To our knowledge, we are the first to report a diminished glycolytic activity of human dendritic cells upon C-type lectin stimulation. Overall, our findings highlight the impact of tumor-associated glycans on dendritic cell biology and metabolism and will increase our understanding on how glycans can shape immunity.

## Introduction

Expression of tumor-associated glycan structures has emerged as one of the hallmarks of cancer, and is associated with many of the pathological steps in tumor progression (1). Such tumor-associated glycans can be recognized by C-type lectin receptors expressed on immune cells. Especially, the macrophage galactose-type lectin receptor (MGL, CD301) has a high preference for tumor-associated glycans and can particularly recognize terminal *N*-acetylgalactosamine (α- or β-linked GalNAc) residues, as found for instance in the truncated *O*-glycan Tn antigen (αGalNAc-Ser/Thr), which is predominantly expressed on many cancer cells, including, colorectal, cervical and breast cancer cells, and has been associated with metastasis and poor prognosis (2–5). MGL is the only C-type lectin receptor within the human immune system that interacts with sialylated and non-sialylated Tn antigen, (asialo)GM2 (GalNAcβ1-4Gal), and the LacdiNAc epitope (GalNAcβ1-4GlcNAc) (6–11). As a consequence, MGL can discriminate between the healthy and tumor-derived glyco-forms of the mucin MUC1 (12).

MGL is exclusively expressed by human dendritic cells (DCs) and macrophages (Mφs) (9, 13, 14). MGL levels are increased on tumor-associated DCs and Mφs, as well as on tolerogenic DCs (5, 12, 14), which are able to dampen T cell immunity in a MGL-dependent manner through the interaction with Tn antigen on CD45 molecules of activated effector T cells. This results in decreased T cell proliferation, reduced inflammatory cytokine production and increased T cell apoptosis (15). Tn antigen- or antibody-mediated activation of MGL on monocyte-derived DCs (moDCs) and CD1c^+^ blood DCs increases TLR-mediated IL-10 and TNF-α secretion (9, 16), and induces the formation of suppressive IL-10 producing CD4^+^ T cells (17). In addition, MGL ligation decreases allogenic CD4^+^ T cell proliferation in a IL-10 dependent manner (18). Together, these findings highlight the potential of MGL to modulate immune responses, however, the impact of MGL-glycan interactions on DC function is still not completely understood.

Recently, we identified a secondary binding site in the MGL molecule (19). While the primary binding site engages the GalNAc monosaccharide, this secondary binding site was essential for binding of the peptide backbone of Tn-containing glycopeptide ligands and for the binding of GalNAc epitopes on tumor cell lines (19). Moreover, binding of different terminal α- or β-linked GalNAc ligands to MGL induces strong and unique alterations in the MGL molecular conformation, depending on the presentation and context of the GalNAc moiety (11). This suggests that MGL triggering can result in the activation of different signaling pathways, and thereby differentially affect biological processes, depending on the MGL ligand used. Furthermore, it emphasizes the importance to use multiple MGL ligands and to dissect ligand-specific binding and subsequent biological effects in future research. Indeed, MGL engagement can trigger the activation of a multitude of signaling cascades, involving the ERK/MAPK, NF-κB, PI3K-Akt, and/or PLCγ2 pathways, although findings on the activity of these signaling pathways upon MGL stimulation are often contradicting (1, 6, 9, 18, 20). This may, thus, depend on the actual ligand- or antibody used to induce conformation of the MGL molecule and warrants further investigation into the ligand-specific signaling induced after binding the MGL receptor.

During the last decades, it became clear that changes in the immunological function of APCs are strongly associated with metabolic alterations important to adapt to new cellular requirements (21–26). In resting DCs, fatty acid oxidation and low levels of glycolysis are the main drivers of mitochondrial respiration, and products of glycolysis pathway are fully catabolized during respiration. Upon TLR activation or under hypoxic conditions, the metabolic activity of DCs changes toward increased glycolytic activity, while mitochondrial respiration is unaffected or even decreases (21–23, 26). The metabolic shift upon TLR activation seems to support the increased demand of *de novo* fatty acid and protein synthesis required for ER and Golgi expansion, optimal antigen presentation, and protein (cytokine) secretion (23). Moreover, the augmentation of glycolytic activity is essential for proper DC activation, as blocking glycolysis strongly inhibits DC cytokine production, expression of co-stimulatory markers and the induction of T cell proliferation (21–23, 26). However, compared to unstimulated and TLR-stimulated DCs, tolerogenic DCs display an increased mitochondrial activity, while the glycolytic rate of LPS-stimulated tolerogenic DCs was completely abolished (27). Also in Mφ subtypes the metabolic activity seems to be differentially regulated. Whereas, the more pro-inflammatory M1-type Mφs have a high glycolytic activity and are interrupted in their TCA cycle at two distinct sites, the more resolving M2-type Mφs mainly rely on a fully active TCA cycle (28–30).

Although these recent publications provide us with knowledge on the effect of TLR stimulation, hypoxia and tolerogenic compounds on metabolic reprogramming of DCs, the impact of tumor-associated glycans nor MGL ligation on DC metabolism has never been investigated before. In the present study, we generated glycodendrimers exposing two different MGL ligands, containing either terminal α-linked GalNAc residues or extended β-linked GalNAc residues. These two different MGL ligands were selected based on their ligand-specific binding capacities for MGL, in which only the extended β-linked GalNAc structure requires the secondary binding site for efficient binding to the MGL receptor. The impact of MGL ligation on human moDC biology was investigated using RNA sequencing analysis, followed by GO term enrichment and pathway analysis. Our findings highlight the potential of C-type lectin receptors to fine-tune not only DC cytokine and T cell responses, but also to shape DC metabolism as an effector mechanism.

## Materials and Methods

### Glycodendrimer Synthesis

The generation 2.0 PAMAM dendrimers with a cystamine core (Sigma-Aldrich, cat#647829) were conjugated to three different glycans via reductive amination using the free reducing ends of the glycans and the dendrimer arm amino moieties. Approximately 20 equivalents of α-D-*N*-Acetylgalactosaminyl 1–3 galactose (Dextra Laboratories UK, cat#G283), asialoGM2 ganglioside sugar-*N*-Acetyl-propargyl (Elicityl, cat#GLY104), and D-(+)-Galactose (Sigma-Aldrich, cat#G0750) per dendrimer were dissolved in Dimethylsulphoxide (DMSO) and acetic acid (8:2) to generate the αGalNAc, GalNAcβ1-4Gal and control dendrimers, respectively. Per dendrimer 160 equivalents of the 2-Methylpyridine borane complex (Sigma-Aldrich, cat#65421) were added to a total volume of 200 μL. The reaction was incubated at 65°C for 2 h with frequent vortexing. The reaction products were purified over disposable PD10 desalting columns (GE Healthcare, cat#GE17-0851-01) in 50 mM Ammonium Formate pH 4.4 (NH_4_HCO_3_). Multiple lyophilization cycles retrieved the glycodendrimers, whereafter the products were validated using LC-MS.

### Functional Binding Assays

NUNC maxisorp plates (Thermo Scientific) were coated overnight at room temperature with glycodendrimers using 0.05 M NaHCO_3_ pH 9.7. Coated plates were washed with TSM (20 mM Tris–HCl pH 7.4, containing 150 mM NaCl, 1 mM CaCl_2_, and 2 mM MgCl_2_) and blocked for 30 min at 37°C with TSM containing 1% BSA. Plates were washed with TSM and incubated for 2 h at room temperature with 1 μg/ml of the biotinylated *Helix pomatia* agglutinin (HPA, Sigma, cat#L6512), 1 μg/ml biotinylated *Vicia villosa* lectin (VVL, Vector laboratories, cat#B-1235), 0.5 μg/ml MGL-Fc (7), or 0.5 μg/ml the mutant MGL H259T-Fc (19) in TSM containing 1% BSA. Plates were washed with TSM containing 0.05% Tween-20 and binding was detected using peroxidase-labeled streptavidin (Biosource, cat#SNN2004) or peroxidase-labeled goat anti-human IgG-Fc (Jackson ImmunoResearch, cat#109-036-098). TMB was used as a substrate to visualize binding and the reaction was stopped with 0.8 M H_2_SO_4_. Optical densities were measured at 450 nm on iMark Microplate Absorbance Reader (Bio-Rad).

### Dendritic Cell Cultures

Monocytes were isolated from buffy coats of healthy volunteers upon informed consent (Sanquin, Amsterdam, The Netherlands) using Ficoll and subsequent Percoll gradient. Monocytes were stimulated as described below or further differentiated into dendritic cells (DCs) in RPMI-1640 medium, supplemented with 10% FCS, Penicillin/streptomycin (Lonza, cat#DE17-602E, 100 U/ml), L-Glutamine (Lonza, cat#BE17-605E, 2 mM), IL-4 (Immunotools, cat#11340047, 12.5–25 ng/ml), GM-CSF (Immunotools, cat#11343127, 12.5–25 ng/ml) for 4 days at 37°C, 5% CO_2_. Expression of MGL was determined by incubating the cells with anti-CD301-PE antibody (Miltenyi Biotec, cat#130-109-641, RRID: AB_2657159) for 30 min at 4°C and analyzed using CyAn ADP High-Performance Flow Cytometer (Beckman Coulter) and FlowJo software v10 (BD Biosciences). DAPI (4′,6-Diamidine-2′-phenylindole dihydrochloride, Invitrogen, cat#D3571, 200 ng/ml) was added to exclude dead cells from the analysis.

### Monocyte and Dendritic Cell Stimulations

To stimulated monocytes and moDCs with glycodendrimers, 1 μM of control, αGalNAc or GalNAcβ1-4Gal glycodendrimers were coated overnight at room temperature using 0.05 M NaHCO_3_ pH 9.7 and sterile 96-wells NUNC Maxisorp plates. Next, plates were washed with PBS and seeded with 0.5–1·10^5^ monocytes or day 4 moDCs in RPMI-1640, supplemented with 10% FCS, L-Glutamine (2 mM), and penicillin/streptomycin (100 U/ml) and incubated at 37°C, 5% CO_2_. To block the MGL receptor, a mixture of two anti-MGL blocking antibodies [1G6.6 (14) and anti-CD301 (Dendritics, cat#DDX0010P-100, clone: 25A10.03)] was added 45 min prior to glycodendrimer stimulation at a concentration of 150 μg/ml. The final concentration of MGL blocking antibodies during culture was 15 μg/ml. Lipopolysaccharide (LPS, E. coli 0111:B4, Sigma-Aldrich, cat#L4391, 10 ng/ml) was added as additional stimuli where indicated. For LPS challenge, 10 ng/ml LPS was added during the last hour of moDC culture before the start of extracellular flux analyses.

### Cytokine Production

After overnight stimulation with glycodendrimers, supernatants were collected and IL-10 production was determined with the LEGENDplex Multi-Analyte Flow Assay Kit, according to manufacturer's instructions (Biolegend, Human inflammation Panel 1, cat#740809) or with an enzyme linked immunosorbent assay (ELISA) using the following antibodies and standard: 0.5 μg/ml IL-10 capture antibody (Thermo Fisher Scientific, cat# 14-7108-85, RRID: AB_468439), 0.25 μg/ml IL-10 detection antibody (Thermo Fisher Scientific cat# 13-7109-85, RRID: AB_466921) and recombinant human IL-10 as a standard (BD Biosciences, cat#554611).

### mRNA Library Preparation

Dendritic cells were harvested and washed with ice-cold PBS after 4 h of dendrimer stimulation, and pooled to obtain sufficient amounts of RNA. Total RNA was extracted with a standard TRIzol isolation protocol (Thermo Fisher Scientific, cat#15596018). Quantity and purity was tested using the Nanodrop-2000 spectrophotometer (Nanodrop Technologies). To prepare the mRNA library, 2 μg RNA per sample was used. The library was synthesized using the TruSeq® Stranded mRNA Sample preparation kit (Illumina, cat# RS-122-9004), according to manufacturer's LS protocol. The product quality during library generation was analyzed on the Agilent 2100 Bioanalyzer using the DNA 7500 chip (Agilent Technologies, cat# 5067-1506).

### RNA Sequencing, Alignment, and mRNA Expression Analysis

The library was sequenced on the HiSeq2500 instrument (Illumina) with a single read type of 50 bp (Tumor Genome Analysis Core, VUmc, Amsterdam, The Netherlands) using standard Illumina protocols. RNA sequencing reads were quality trimmed using Sickle (31) and quality checked using FASTQC (https://www.bioinformatics.babraham.ac.uk/projects/fastqc/) (32). Reads were aligned to the Ensemble human genome GRCh38.p10 (release 90) using Spliced Transcripts Alignment to a Reference (STAR 2.5.4a) (33) and subsequent Sequence Alignment Map (SAM) files were created (34). FeatureCounts (R package Subread 1.26.1, R 3.4.0) (35, 36) was used to quantify aligned reads, excluding multimapping and multi-overlap reads. R package edgeR (version 3.18.1) (37, 38) was subsequently used for library size adjustment, trimmed mean of M-values (TMM) normalization and analysis. Multidimensional scaling (MDS) plots were used to visualize sample distribution among different treatments. Due to the high degree of donor variation visible in the MDS plots, as well as clustering on LPS, a generalized linear model (GLM) was created to identify the significant differentially expressed genes (DEGs) (Likelihood ratio test with Benjamini-Hochberg correction, false discovery rate (FDR) <0.05). The GLM model included the following factors: donor, LPS, treatment and interaction effects, and a forward selection method was selected giving the lowest dispersion for the model. Sequencing data is publicly available at the Sequence Read Archive (SRA) Gene Expression Omnibus through GEO Series accession number GSE143699.

### Gene Ontology Term Enrichment and Pathway Analysis

Gene Ontology (GO) term enrichment analysis was performed on the 1106 DEGs in GalNAcβ1-4Gal stimulated moDCs using Cytoscape v3.7.1 (https://cytoscape.org/) (39), and the ClueGO plugin (v2.5.4) (http://apps.cytoscape.org/apps/cluego) (40). Significantly enriched GO terms (Benjamini-Hochberg corrected FDR <0.05) were determined with the ontology source GO_BiologicalProcess-EBI-UniProt-GOA, and were subsequently visualized using view style Groups, GO level 6–13, and a kappa score threshold of 0.45. Where indicated, GO term-associated DEGs were extracted and subjected to heatmap visualization using Morpheus (https://software.broadinstitute.org/morpheus/). Pathway based data integration and visualization was performed using Pathview Web server under default settings (https://pathview.uncc.edu/) (41, 42).

### Glucose Uptake and Mitochondrial Membrane Potential

Glucose uptake was determined after overnight stimulation of moDCs with glycodendrimers as described above. Dendritic cells were washed twice and starved from glucose for 1 h in DMEM medium without glucose and phenol red (ThermoFisher, cat# A14430), supplemented with 10% FCS, Penicillin/streptomycin (100 U/ml), L-Glutamine (2 mM), and sodium pyruvate. 2-NBDG (2-Deoxy-2-[(7-nitro-2,1,3-benzoxadiazol-4-yl)amino]-D-glucose, Sigma-Aldrich, cat#72987, 25 μM) was added for 90 min at 37°C, after which moDCs were washed with PBS. To measure the mitochondrial membrane potential, moDCs were washed two times and stained for 20 min at 37°C with TMRM (Tetramethylrhodamine methyl ester perchlorate, Sigma-Aldrich, cat#T5428, 50 nM) and subsequently washed with PBS. DAPI was added to exclude dead cells from the analysis. DCs were analyzed using CyAn ADP High-Performance Flow Cytometer and FlowJo software v10 (BD Biosciences).

### Metabolic Extracellular Flux Analysis

Dendritic cells (50.000) were stimulated overnight with glycodendrimers as described above, either in the presence or absence of LPS or anti-MGL blocking antibodies. Seahorse XF96 Cell culture microplates (Agilent, cat#101085-004) were coated for 1-2 h with poly-L-Lysine (Sigma-Aldrich, cat#P8920 or P4707), after which plates were washed with PBS. moDCs were harvested, washed and transferred to the Seahorse Cell Culture Microplate, followed by a short spin at 500 rpm and 1–1.5 h incubation at 37°C in Seahorse XF Base medium (Agilent Technologies, cat#103335-100), supplemented with 2 mM L-Glutamine (Sigma-Aldrich, cat#G8540), 1 mM Sodium Pyruvate (Sigma-Aldrich, cat#P5280), 2 mM sodium bicarbonate (Sigma-Aldrich, cat#S6014), 5 mM HEPES (Gibco, cat#15630-056), and set to a pH of 7.4–7.6. For LPS challenge, 10 ng/ml LPS was added to the culture medium. Extracellular acidification rates (ECAR) and oxygen consumption rates (OCR) were measured after 1 h on the Seahorse XF96 Flux Analyzer (Agilent) according to manufacturer's protocols (cat#103016-400 and 103020-400). Two injection strategies were used: 10 mM Glucose (Glc, Sigma-Aldrich, cat#G7021)/ 1.5 μM Oligomycin (OM, Sigma-Aldrich, cat#O4876)/ 50 mM 2-Deoxy-D-Glucose (2-DG, Carbosynth, cat#MD05187) or 10 mM Glucose/ 1.5 μM OM/ 0.5 μM Trifluoromethoxy carbonylcyanide phenylhydrazone (FCCP, Sigma-Aldrich, cat#C2920)/ 0.5 μM Rotenone/Antimycin A (Rot/AA, Sigma-Adrich, cat#R8875 and cat#A8674).

Data was normalized using the DC protein Assay Kit, according to manufacturer's protocols (Biorad, cat#5000111). Changes in ECAR in response to Glc and OM injections were used to calculate glycolysis, glycolytic capacity and glycolytic reserve. Changes in OCR in response to OM, FCCP, and Rot/AA were used to calculate basal respiration, ATP-linked respiration and maximal respiration. Basal ECAR/OCR was measured after Glc injection.

### Statistics

Results were analyzed for statistical significance in GraphPad Prism v8.0.2 using paired nonparametric one-way ANOVA, comparing the αGalNAc and GalNAcβ1-4Gal conditions to the control condition (^*^p < 0.05, ^**^p < 0.01).

## Results

### Generation of Glycodendrimers Exposing Two Different MGL Ligands

**Figure 1:**
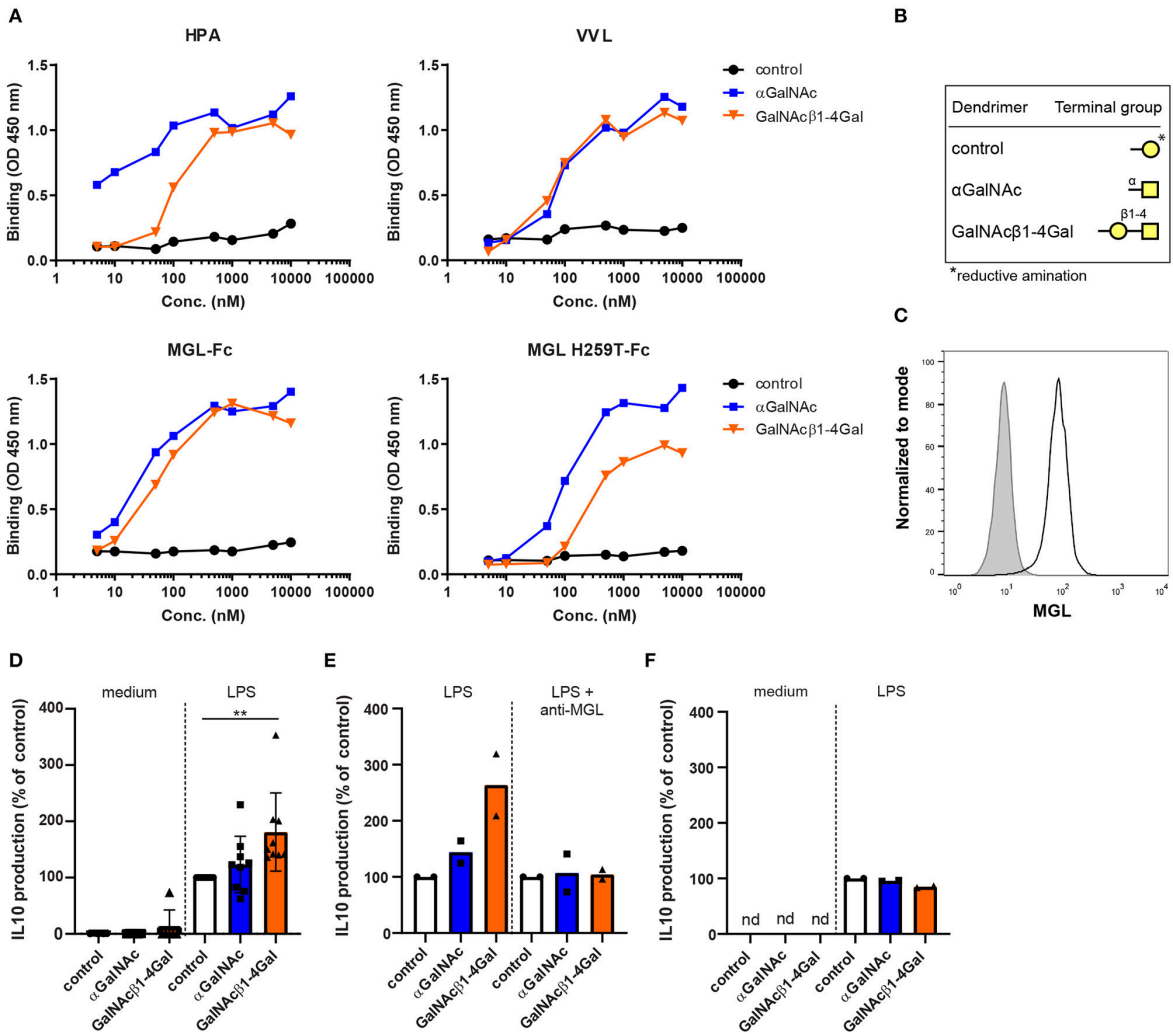
Ligand-specific binding of terminal GalNAc glycodendrimers to MGL enhances LPS-induced IL-10 production in human moDCs. **(A)** Binding of titrated αGalNAc (blue squares) or GalNAcβ1-4Gal (orange triangles) glycodendrimers (x-axis) to the plant lectins *Helix pomatia* agglutinin (HPA) and *Vicia villosa* lectin (VVL) (upper panels), and to MGL-Fc and MGL H259T-Fc (lower panels). Binding was measured using an ELISA-based assay and was detected at an optical density of 450 nm. Binding of control glycodendrimers was used as a negative control (black circles). **(B)** Terminal glycan structures of the generated control, αGalNAc and GalNAcβ1-4Gal glycodendrimers. ^*^The ring form of galactose, used to generate the control glycodendrimers, was opened up during glycodendrimer generation upon reductive amination. Galactose (yellow circle), *N*-acetyl-D-galactosamine (yellow square). **(C)** Unstimulated monocyte-derived dendritic cells (moDCs) were analyzed for MGL expression (black line) after 4 days of differentiation using flow cytometry. Isotype control is depicted in gray. One representative of three independent experiments is shown. **(D)** moDCs were stimulated overnight with control (white), αGalNAc (blue), or GalNAcβ1-4Gal (orange) glycodendrimers in the absence or presence of LPS after which IL-10 production was measured in the supernatants. Relative secretion compared to moDCs stimulated with LPS and control glycodendrimers is depicted (set to 100%). Error bars represent standard deviation of six or nine independent experiments for moDCs and LPS-stimulated moDCs, respectively. **(E)** moDCs were stimulated overnight with LPS and control (white), αGalNAc (blue), or GalNAcβ1-4Gal (orange) glycodendrimers in the absence or presence of anti-MGL blocking antibodies. IL-10 production was measured in the supernatants. Relative secretion compared to moDCs stimulated with control glycodendrimers is depicted (set to 100%). **(F)** Monocytes (*n* = 2) were stimulated overnight with the three different glycodendrimers in the absence or presence of LPS as described for moDCs **(D)**. (nd = not detectable).

To investigate the effect of MGL ligation on moDC biology, we generated control dendrimers and two different glycodendrimers exposing the MGL ligands αGalNAc or GalNAcβ1-4Gal (Figure 1B). We choose these ligands based on their differential binding to the secondary binding site present in the MGL carbohydrate recognition domain (11, 19). To validate the αGalNAc and GalNAcβ1-4Gal glycodendrimers, lectin binding assays were performed using *Helix pomatia* agglutinin (HPA) and *Vicia villosa* lectin (VVL). Although both HPA and VVL can recognize α- and β-GalNAc, HPA has a clear preference for αGalNAc moieties (43). As expected, VVL bound both glycodendrimers with the same affinity, also confirming equal coating of both glycodendrimers, whereas HPA binding to the αGalNAc glycodendrimers was stronger compared to the GalNAcβ1-4Gal glycodendrimers (Figure 1A).

We next addressed whether our glycodendrimers could bind MGL and its H259T mutant. We have previously shown that the MGL H259T mutant has a strongly reduced affinity for Tn antigen and is unable to engage elongated MGL ligands, such as the GalNAcβ1-4Gal moiety (19). Indeed, both the αGalNAc and GalNAcβ1-4Gal glycodendrimers were recognized by the wild type MGL-Fc (Figure 1A). In contrast, the H259T mutation reduced the binding capacity of MGL H259T-Fc to the GalNAcβ1-4Gal glycodendrimer about 10-fold, while the binding to αGalNAc was unaltered, as this epitope only engages the primary binding site in the MGL molecule (Figure 1A). Thus, the two GalNAc containing glycodendrimers are both recognized by MGL, whereas only the GalNAcβ1-4Gal glycodendrimer requires the secondary binding site of MGL for efficient binding to the receptor.

Recognition of Tn antigen structures by MGL-expressing moDCs is known to increase TLR-induced IL-10 production (16, 17). To investigate whether our glycodendrimers could also evoke IL-10 secretion, we stimulated MGL^pos^ human moDCs with the three different glycodendrimers both in the presence and absence of the TLR4 ligand LPS (Figure 1C), and analyzed IL-10 secretion in the supernatants. MGL ligation did not induce IL-10 production in the absence of LPS, which is in line with previous observations (Figure 1D) (16). Strikingly, only the GalNAcβ1-4Gal dendrimers were able to induce IL-10 production in the presence of LPS, suggesting that the two dendrimers tested might trigger different signaling cascades in the moDC (Figure 1D). GalNAcβ1-4Gal-induced IL-10 production was dependent on MGL receptor signaling, as the GalNAcβ1-4Gal-induced IL-10 secretion was abrogated in the presence of MGL blocking antibodies (Figure 1E). Moreover, GalNAcβ1-4Gal dendrimers did not induce IL-10 production in MGL^neg^ human monocytes (Figure 1F) (13).

### GalNAcβ1-4Gal Reduces Expression of Key Enzymes Involved in Energy Metabolism

**Figure 2:**
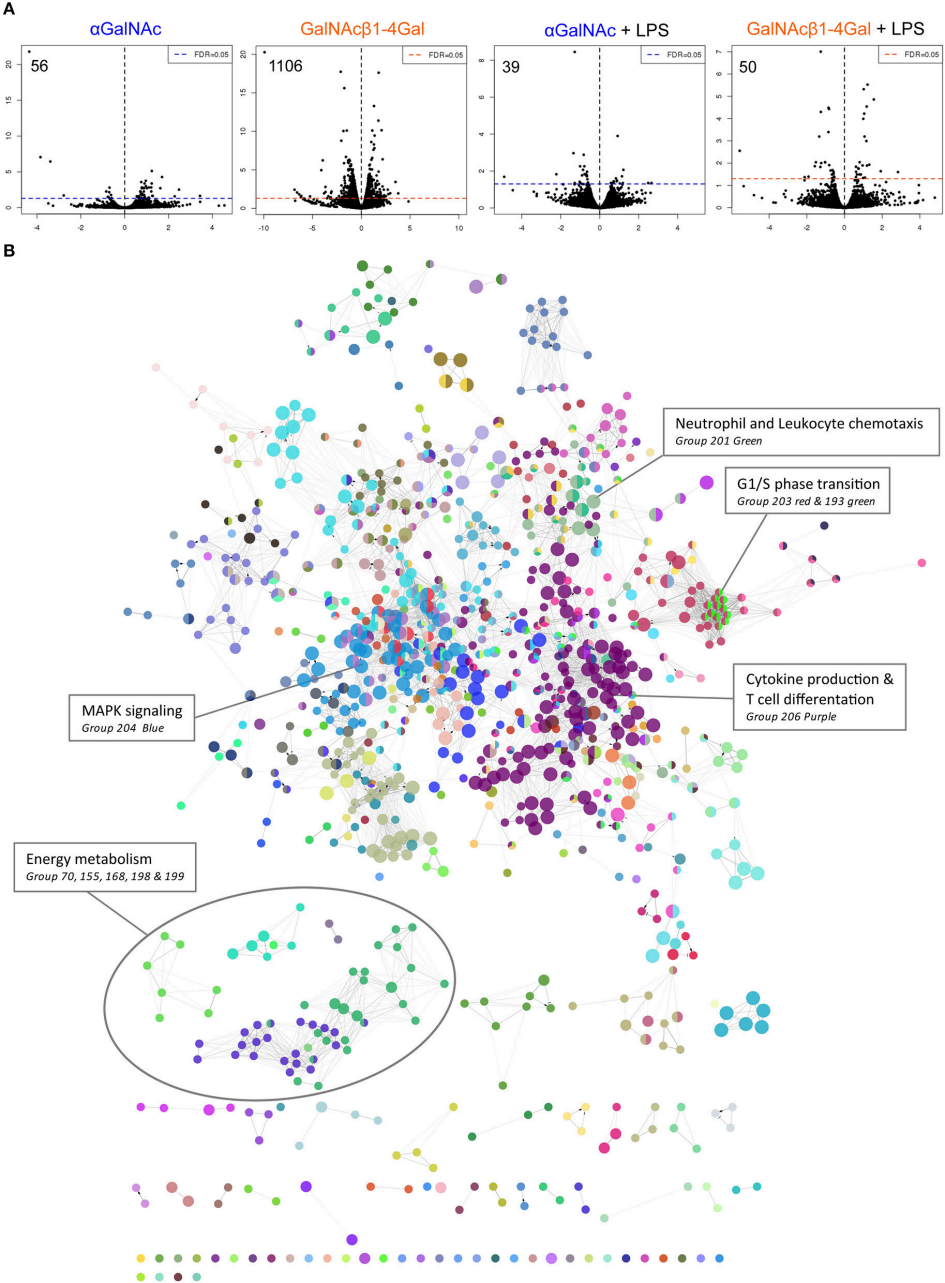
GalNAcβ1-4Gal stimulation strongly alters moDC transcriptional activity. **(A)** Gene expression analysis of moDCs stimulated with αGalNAc (blue) or GalNAcβ1-4Gal (orange) glycodendrimers for 4 h in the presence or absence of LPS. Depicted are the fold change (x-axis) and FDR-adjusted *p*-values in a minus log 10 transformation (y-axis). **(B)** GO term enrichment analysis was performed on the 1106 differentially expressed genes (DEGs) of GalNAcβ1-4Gal-stimulated moDCs. Gene sets defined by significantly enriched GO terms were visualized as nodes, connected lines represent overlapping genes between gene sets. Coloring was based on GO groups defined by the software. Results of the GO term enrichment analysis are also listed in Table S5.

To investigate the effect of MGL ligation at the transcriptional level, moDCs from three donors were stimulated with control, αGalNAc or GalNAcβ1-4Gal glycodendrimers (further referred to as αGalNAc or GalNAcβ1-4Gal) in the absence or presence of LPS, followed by RNA sequencing analysis. Differential expression analysis revealed that MGL stimulation by GalNAcβ1-4Gal in the absence of LPS strongly affected the transcriptional profile in moDCs, yielding increased expression of 378 genes, and reduced expression of 728 genes (1106 DEGs in total; Figure 2A, Table S2), even though the MGL-mediated induction of IL-10 requires concomitant TLR stimulation (16). In contrast, αGalNAc stimulation had only a minimal effect on gene expression, affecting the expression of 56 genes in total (Figure 2A, Table S1). In the presence of LPS, the effect of MGL ligation on gene expression was much smaller, yielding 39 DEGs for αGalNAc and 50 DEGs for GalNAcβ1-4Gal (Figure 2A, Tables S3, S4).

Because we only obtained a substantial amount of DEGs after the GalNAcβ1-4Gal stimulation, we continued the analysis only with this gene set to assess the effect of MGL ligation on moDC biology. These 1106 DEGs were subjected to Gene Ontology (GO) term enrichment analysis (Table S5, Figure 2B). Among the significant GO terms identified, many terms involved MAPK signaling, including ERK1/2, JNK and p38 signaling (Group76 and 204 in Figure 2B, Figure S1 and Table S5), as described previously (1, 6, 18). In addition, an effect of MGL triggering on NF-κB signaling (group 117, Table S5) (20) and T cell differentiation could be confirmed (Group 206, Figure 2B, Table S5) (17, 18). The GO term enrichment analysis, furthermore, suggested that stimulation with GalNAcβ1-4Gal alters G1/S phase transition and neutrophil and leukocyte chemotaxis (Group 193, 201, and 203, Figure 2B, Table S5).

**Figure 3:**
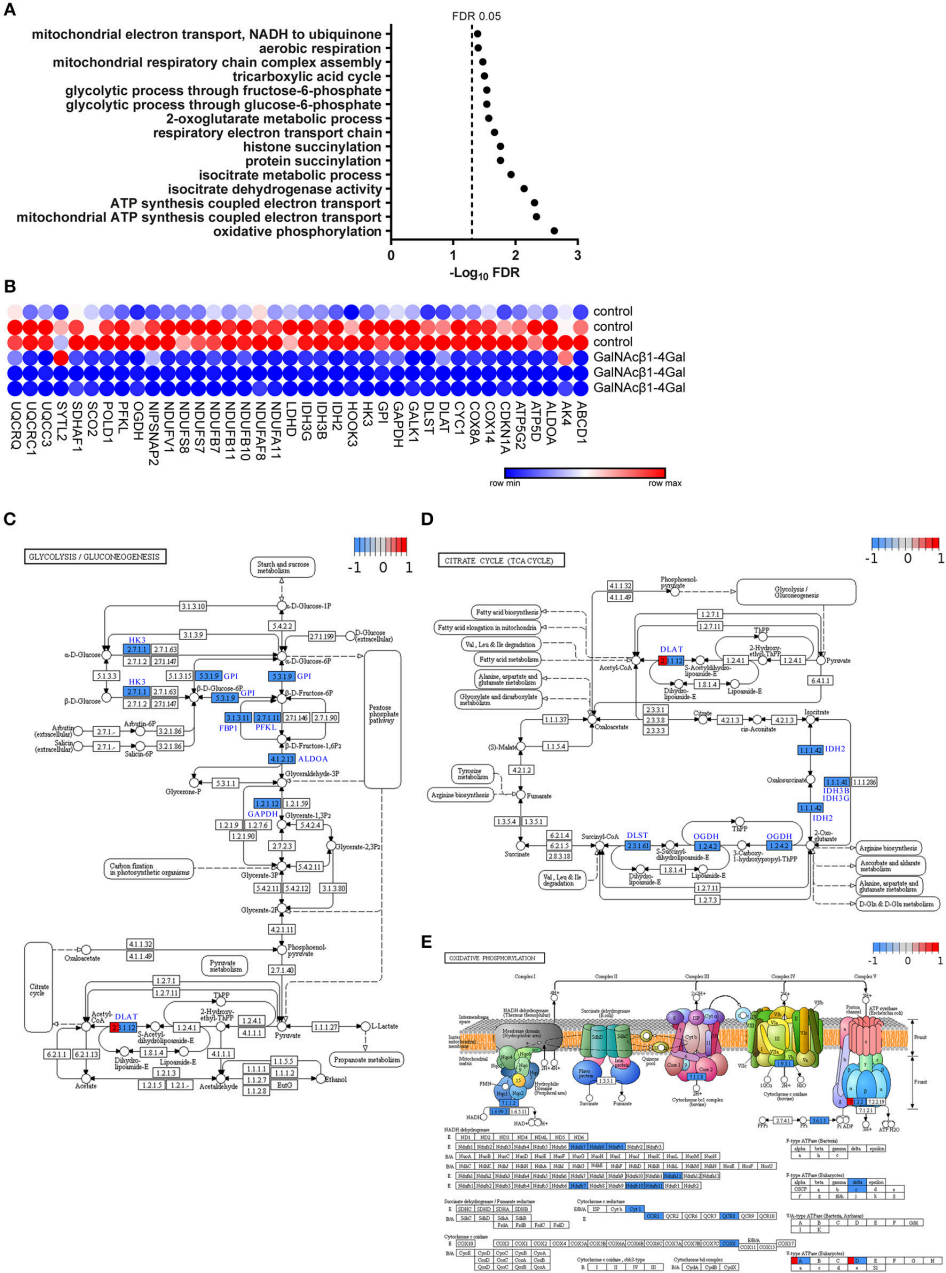
GalNAcβ1-4Gal reduces the expression of key enzymes involved in the glycolysis pathways, TCA cycle and oxidative phosphorylation in moDCs. **(A,B)** Significant GO terms **(A)** and extracted DEGs **(B)** of the 1106 DEGs in GalNAcβ1-4Gal-stimulated moDCs are depicted for the GO groups on glycolysis (group 70), TCA cycle (Group 168) and oxidative phosphorylation (Group 155) (Table S5). **(A)** FDR-adjusted *p*-values were plotted in a minus log 10 transformation. **(C–E)** Pathway analysis and visualization of the DEGs in GalNAcβ1-4Gal stimulated moDCs for Glycolysis/Gluconeogenesis **(C)**, TCA Cycle **(D)**, and Oxidative phosphorylation **(E)**.

Interestingly, an enrichment in GO terms associated with energy metabolism could be observed in GalNAcβ1-4Gal-stimulated moDCs (Figure 2B). This cluster of GO terms includes terms on glycolysis, oxidative phosphorylation, tricarboxylic acid (TCA) cycle (Group 70, 155, 168 in Figure 2B, Figure 3A, Table S5), and nucleoside biosynthesis (Group 198 and 199 in Figure 2B, Table S5). Strikingly, expression of all DEGs associated with glycolysis, oxidative phosphorylation, and the TCA cycle, were reduced upon GalNAcβ1-4Gal stimulation (Figure 3B). This included the expression of key enzymes involved in glycolysis pathway, such as *Hexokinase 3* (*HK3*), *Glucose-6-Phosphate Isomerase* (*GPI*), *Glyceraldehyde-3-Phosphate Dehydrogenase* (*GAPDH*), and *Fructose-Bisphosphate Aldolase A* (*ALDOA*), as well as key enzymes involved in the TCA cycle and oxidative phosphorylation, such as *Isocitrate Dehydrogenases* (*IDH2, IDH3B, IDH3G*), and *NADH dehydrogenases* (*NDUF* genes) (Figure 3B). None of these GO terms were enriched in αGalNAc-stimulated moDC, nor did we observe differential expression of any of these metabolic genes in αGalNAc-stimulated moDC (data not shown). Pathway analysis of the DEGs, furthermore, revealed that MGL triggering by GalNAcβ1-4Gal affected genes along the entire pathway of glycolysis and oxidative phosphorylation (Figures 3C–E). Thus, stimulation of moDCs with the MGL-binding GalNAcβ1-4Gal glycodendrimers appears to have a strong suppressive effect on energy metabolism.

### MGL Ligation Suppresses the Glycolytic Activity in moDC

**Figure 4:**
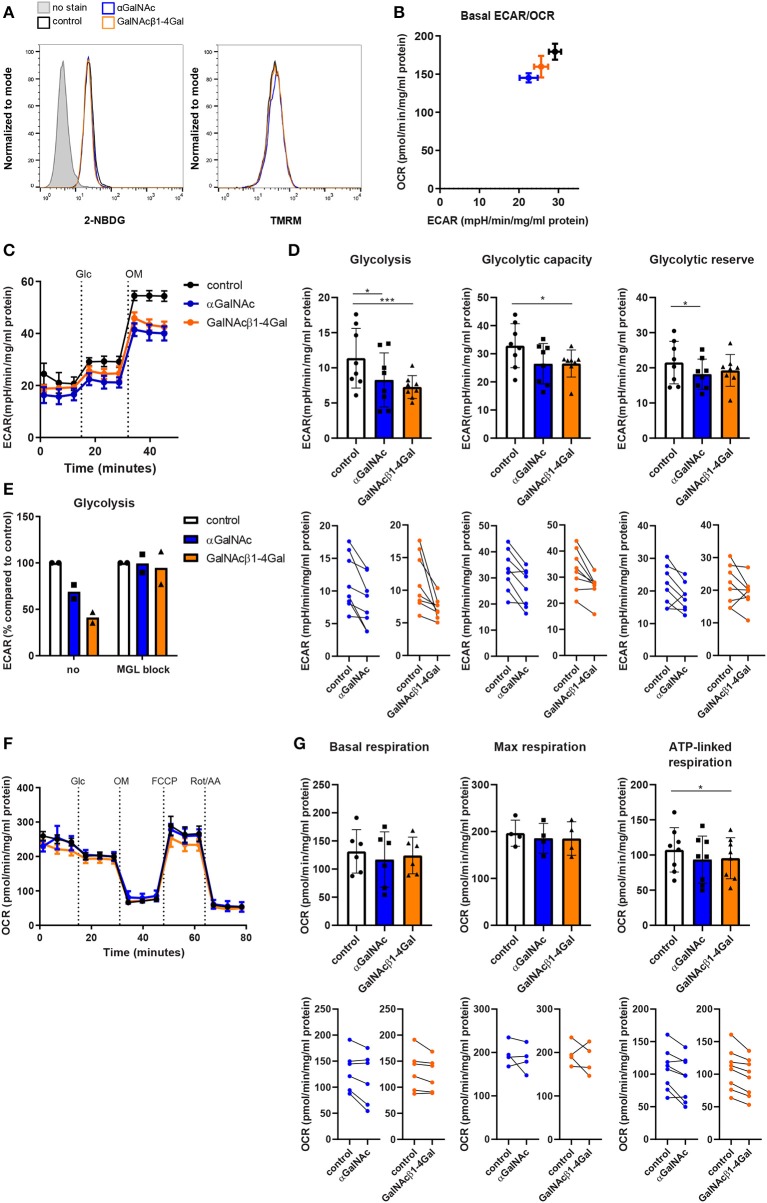
MGL ligation suppresses the glycolytic activity in moDCs. **(A)** Glucose uptake (2-NBDG staining, left) and the mitochondrial membrane potential (TMRM staining, right) of moDCs stimulated overnight with αGalNAc (blue), GalNAcβ1-4Gal (orange), or control (black) glycodendrimers. Data was analyzed by flow cytometry. One representative of 2–4 independent experiments is shown. **(B–G)** moDCs were stimulated overnight with αGalNAc (blue), GalNAcβ1-4Gal (orange), or control (black) glycodendrimers and subsequently subjected to extracellular flux analysis. Data was normalized based on protein content. **(B)** Basal extracellular acidification rate (ECAR) and oxygen consumption rate (OCR) were measured after glucose addition. One representative of five independent experiments is depicted. **(C)** During extracellular flux analysis, ECAR in response to treatment with Glucose (Glc) and Oligomycin (OM) was measured. One representative of eight independent experiments is depicted. Error bars represent standard deviation of 4–6 technical replicate measurements. **(D)** Glycolysis, glycolytic capacity, and glycolytic reserve were determined using the ECAR levels before and after treatment with Glc and OM (*n* = 8). Individual donors are depicted in the line graphs (lower panels). **(E)** Glycolysis was determined in moDCs stimulated with the different glycodendrimers both in the absence or presence of anti-MGL blocking antibodies. ECAR of moDCs stimulated with control glycodendrimers was set to 100%. **(F)** During extracellular flux analysis, OCR in response to treatment with Glc, OM, FCCP, and rotenone plus antimycin A (Rot/AA) was measured. One representative of six independent experiments is depicted. Error bars represent standard deviation of 4-6 technical replicate measurements. **(G)** Basal respiration, maximal respiration and ATP-linked respiration were determined using the OCR levels before and after treatment with OM, FCCP and Rot/AA. Error bars represent standard deviation of 4–8 independent experiments. Individual donors are depicted in the line graphs (lower panels). *p < 0.05; ***p < 0.001.

To confirm whether the decreased expression of key enzymes involved in glycolysis and oxidative phosphorylation actually results in changes in the metabolic activity of moDCs, we treated moDCs with the GalNAcβ1-4Gal glycodendrimers and also included the αGalNAc dendrimers to evaluate whether the effects on metabolism would be GalNAcβ1-4Gal-specific. αGalNAc and GalNAcβ1-4Gal stimulated moDCs were starved from glucose and subsequently labeled with either a fluorescent glucose agonist (2-NBDG) or subjected to metabolic extracellular flux (Seahorse XF) analysis (Figure 4). In addition, we measured the mitochondrial activity of αGalNAc- and GalNAcβ1-4Gal-stimulated moDCs using TMRM staining. Glucose uptake and the mitochondrial membrane potential of moDCs were not affected upon MGL ligation (Figure 4A). However, both αGalNAc and GalNAcβ1-4Gal stimulated moDCs showed a reduction in the basal extracellular acidification rate (ECAR) after overnight stimulation (Figures 4B,C). Moreover, the glycolytic activity was significantly decreased in both αGalNAc- and GalNAcβ1-4Gal-stimulated moDCs, in a MGL-dependent manner (Figures 4D,E). Although a reduction in the glycolytic reserve and glycolytic capacity of αGalNAc and GalNAcβ1-4Gal stimulated moDCs could be observed in most donors, this effect was significant only in the αGalNAc or GalNAcβ1-4Gal stimulated moDCs, respectively (Figure 4D).

The oxygen consumption rate (OCR), basal respiration and maximal respiration were unaffected upon MGL ligation (Figures 4F,G). ATP-linked respiration in αGalNAc and GalNAcβ1-4Gal stimulated moDC tended to be decreased in most donors, but only reached significance in GalNAcβ1-4Gal stimulated moDC (Figure 4G). Since the glycodendrimers needed to be coated in high absorbance plates to induce MGL signaling, and do not signal when added in solution (16), it was technically not possible to determine the effect of dendrimer stimulation on moDC metabolism in real time.

To address whether reduced glycolytic activity in response to MGL ligation is overruled by strong TLR stimulation, extracellular flux analysis was performed on moDC stimulated with LPS and the different glycodendrimers simultaneously. No effect on glycolytic activity and respiration was observed in DCs when LPS was included in the αGalNAc or GalNAcβ1-4Gal stimulation (Figure S3). Glycan-pretreated moDCs, however, showed reduced glycolytic activity upon subsequent LPS challenge (Figure S3C). Taken together, glycan-based triggering of the MGL receptor affects the metabolic activity of human moDC by reducing their glycolytic activity. In the presence of strong TLR stimulation, however, this inhibitory effect of MGL stimulation on DC metabolism may be overruled dependent on the timing between MGL ligation and TLR ligand exposure.

## Discussion

Immune cell metabolism is becoming more appreciated in the context of immune cell activation, yet the effects of many stimuli, including C-type lectin-glycan interactions, on DC metabolic reprogramming have not been investigated. Here, we demonstrate for the first time the full impact of MGL ligation on DC activation. MGL ligation alters the expression of genes involved in many different biological processes, including MAPK and NF-κB signaling, neutrophil chemotaxis, and G1/S cell cycle transition. In addition, enrichment for GO terms involved in energy metabolism were identified, such as the glycolysis pathway, TCA cycle and oxidative phosphorylation. Strikingly, the expression of all DEGs involved in these pathways was strongly reduced. In addition, we found a new metabolic phenotype, characterized by reduced glycolytic activity in the absence of mitochondrial changes, which to our knowledge has not be reported before in de context of human DC activation.

We employed two different MGL-binding glycodendrimers to study the effect of MGL ligation on DC biology and metabolism. Whereas GalNAcβ1-4Gal increased TLR-induced IL-10 production and affected the expression of 1106 transcripts, αGalNAc had only minimal effects on the transcriptome of DCs and did not alter TLR-induced IL-10 secretion. In addition, a different binding profile was observed between the two different terminal GalNAc structures, in which only GalNAcβ1-4Gal required the secondary binding site of MGL for sufficient binding. This secondary binding site of MGL binds the peptide backbone of Tn-containing glycopeptide ligands, and is essential for the binding of cancer-associated Tn epitopes on tumor cell lines (19). Recent evidence points to ligand-specific conformational changes in the MGL carbohydrate binding domain (11), raising the intriguing possibility that each individual MGL ligand may activate different signaling cascades, and thus a different transcriptional program in the DC. This hypothesis would also explain the differential effects we observed between the two terminal GalNAc structures used in this study. Our findings, furthermore, suggest that binding to both the primary and secondary binding site of MGL enhances the efficiency of MGL signaling, emphasizing the importance of carefully selecting MGL ligands in vaccination or immunomodulatory strategies. For instance, MGL triggering by plate coated anti-MGL strongly induced the activation of many signaling molecules, including ERK1/2, JNK, and CREB (18), whereas these pathways were not activated upon stimulation with soluble MUC1-(T_n_)_2_ constructs (9).

Whereas activation of murine DCs and human monocytes increases their glycolytic activity (21–23, 25), MGL ligation on human DCs decreases the expression of key enzymes involved in glycolysis, the TCA cycle, and oxidative phosphorylation. Furthermore, the glycolytic activity of MGL-stimulated DCs was reduced compared to unstimulated DCs. A decrease in glycolytic activity has not been observed before in human DCs, but has previously been associated with anti-inflammatory, more resolving Mφs displaying protumoral activity (30). These anti-inflammatory Mφs are involved in tissue remodeling and immunosuppression (30). However, the metabolic reprogramming observed in MGL-stimulated DCs does not completely match the metabolic changes observed in these anti-inflammatory Mφs. The mitochondrial activity was increased in anti-inflammatory Mφs compared to unstimulated Mφs (29, 30), whereas we observed no substantial differences in mitochondrial respiration in MGL-stimulated DCs. Apart from reducing the DC glycolytic activity, MGL ligation furthermore supports immature DC tissue retention (44). Therefore, we propose that MGL ligation by carbohydrate ligands, locally expressed in the tissue (44), maintains DC tissue residence and homeostasis in the absence of an infection. In addition, the metabolic program induced upon MGL stimulation may be specific for this C-type lectin receptor, as both mannose receptor and dectin-1 are associated with increased glycolytic activity in human monocytes upon *C. albicans* stimulation (25). Whether similar phenotypic changes are noticeable after glycan-based triggering of MGL^pos^ Mφs is a subject for future studies.

However, in case of an infection, MGL-mediated silencing of immature DCs needs to be overruled to ensure that DCs become activated and perform their effector functions upon pathogenic exposure. This is indeed exactly what happens upon strong pathogenic DC activation, which restores the DCs' migratory capacities (44). Also the reduced glycolytic activity observed in MGL-stimulated DCs is overruled upon prolonged TLR4 stimulation. MGL ligation does, however, induce IL-10 production by DCs in the presence of TLR4 stimulation, and this has been associated with the formation of suppressive CD4^+^ T cells before (17). Together, these data indicate that MGL-primed DCs are reminiscent of a novel tolerogenic metabolic DC phenotype associated with the induction of regulatory T cell responses. A thorough investigation addressing various (tolerogenic) stimuli in combination with metabolic phenotyping may shed new light on our understanding of metabolic changes in tolerogenic or regulatory DCs and Mϕs.

Tumor-associated MGL-ligands, including Tn antigen, are predominantly expressed by many tumors, and have been associated with tumor progression and poor survival in colorectal and cervical cancer patients (4, 5). As many MGL-expressing DCs and Mϕs are infiltrating tumors (5, 12), we postulate that MGL-mediated immune modulation will be most prominent within the context of a tumor. The observed reduction in the glycolytic activity of DCs upon MGL activation might silence a pro-inflammatory state within the DCs, as a strong increase in the glycolytic activity is associated with mature DCs and potent T cell stimulatory capacity (21–23). MGL ligation on DCs will probably reduce the anti-tumor immune response, thereby removing the brake on tumor outgrowth and promoting tumorigenicity.

Different from our *in vitro* culture conditions, DCs can be exposed to hypoxic conditions within the tumor microenvironment (45). Hypoxia induces the expression of hypoxia induced factor 1α (HIF1α), a transcription factor that activates the transcription of glycolytic enzymes and glucose transporters, to increase glycolytic activity (46, 47). A reduced HIF1α expression may be at the basis of our phenotype, for we observed a decreased expression of many genes involved in glycolysis upon MGL ligation. This idea is further supported by our finding that MGL engagement decreases the expression of several signaling molecules involved in HIF-1α translation, such as MEK, ERK and Akt (Figure S2) (21), even though *HIF1*α mRNA levels were unaffected (Table S2). The effect of MGL ligation on DC metabolism within the context of the tumor microenvironment, such as under in the influence of hypoxia, competition for nutrients, and damage associated patterns, remains to be investigated (45). It would be of interest to evaluate whether the increase in glycolytic activity induced under hypoxic conditions would overrule the inhibitory effect of MGL ligation on DC glycolytic activity. Yet, the competition between DCs and tumor cells for specific nutrients (45), such as glucose, may further reduce the glycolytic activity in human DCs, and thereby their anti-tumoral properties.

Taken together, we show that MGL ligation reduces the expression of key enzymes involved in the glycolysis pathway, TCA cycle and oxidative phosphorylation, which results in a reduced glycolytic activity observed in human DCs. The reduced glycolytic activity may further decrease the anti-tumor immune response, however, the impact of MGL stimulation on DCs in the tumor microenvironment needs further investigation. In addition, induction of MGL signaling strongly depends on the nature of the MGL ligand presented, which may be controlled by different binding efficiencies to the secondary binding site in MGL and ligand-specific conformational changes. Apart from the MGL-mediated effects on DC metabolism, GO term enrichment analysis also suggests an role for MGL activation in neutrophil chemotaxis, regulation of cell cycle and several other processes involved in DC activity. Our study, therefore, can also serve as a useful starting point for further investigations on MGL-related changes in DC biology. Our findings highlight the impact of tumor-associated glycans on DC biology and metabolism and will increase our understanding of immune regulation by tumor-associated glycans.

## Data Availability Statement

The datasets generated for this study is publicly available at the Sequence Read Archive (SRA) Gene Expression Omnibus through GEO Series accession number GSE143699 (https://www.ncbi.nlm.nih.gov/geo/).

## Author Contributions

HK and RL generated the GalNAc glycodendrimers. AZ, RL, and JL performed the RNA sequencing and subsequent analysis. AZ, RL, and SB performed the experiments. AZ analyzed all the data. SV supervised the project. AZ and SV wrote the manuscript. YK corrected the manuscript.

### Conflict of Interest

The authors declare that the research was conducted in the absence of any commercial or financial relationships that could be construed as a potential conflict of interest.

## References

[1] Munkley J Elliott DJ. Hallmarks of glycosylation in cancer. Oncotarget. (2016) 7:35478–89. 10.18632/oncotarget.8155 27007155 PMC5085245

[2] Itzkowitz SH Yuan M Montgomery CK Kjeldsen T Takahashi HK Bigbee WL. Expression of Tn, sialosyl-Tn, and T antigens in human colon cancer. Cancer Res. (1989) 49:197–204. 2908846

[3] Schumacher U Adam E. Lectin histochemical HPA-binding pattern of human breast and colon cancers is associated with metastases formation in severe combined immunodeficient mice. Histochem J. (1997) 29:677–84. 10.1023/A:1026404832394 9413741

[4] Lenos K Goos JA Vuist IM den Uil SH Delis-van Diemen PM Belt EJ. MGL ligand expression is correlated to BRAF mutation and associated with poor survival of stage III colon cancer patients. Oncotarget. (2015) 6:26278–90. 10.18632/oncotarget.4495 26172302 PMC4694901

[5] Sahasrabudhe NM van der Horst JC Spaans V Kenter G de Kroon C Bosse T. MGL ligand expression is correlated to lower survival and distant metastasis in cervical squamous cell and adenosquamous carcinoma. Front Oncol. (2019) 9:29. 10.3389/fonc.2019.00029 30761272 PMC6361794

[6] Iida S Yamamoto K Irimura T. Interaction of human macrophage C-type lectin with O-linked N-acetylgalactosamine residues on mucin glycopeptides. J Biol Chem. (1999) 274:10697–705. 10.1074/jbc.274.16.10697 10196140

[7] van Vliet SJ van Liempt E Saeland E Aarnoudse CA Appelmelk B Irimura T. Carbohydrate profiling reveals a distinctive role for the C-type lectin MGL in the recognition of helminth parasites and tumor antigens by dendritic cells. Int Immunol. (2005) 17:661–9. 10.1093/intimm/dxh246 15802303

[8] Mortezai N Behnken HN Kurze AK Ludewig P Buck F Meyer B. Tumor-associated Neu5Ac-Tn and Neu5Gc-Tn antigens bind to C-type lectin CLEC10A (CD301, MGL). Glycobiology. (2013) 23:844–52. 10.1093/glycob/cwt021 23507963

[9] Heger L Balk S Luhr JJ Heidkamp GF Lehmann CHK Hatscher L. CLEC10A is a specific marker for human CD1c(+) dendritic cells and enhances their Toll-like receptor 7/8-induced cytokine secretion. Front Immunol. (2018) 9:744. 10.3389/fimmu.2018.00744 29755453 PMC5934495

[10] Kannagi R Cai BH Huang HC Chao CC Sakuma K. Gangliosides and tumors. Methods Mol Biol. (2018) 1804:143–71. 10.1007/978-1-4939-8552-4_6 29926407

[11] Diniz A Coelho H Dias JS van Vliet SJ Jimenez-Barbero J Corzana F. The plasticity of carbohydrate recognition domain dictates the exquisite mechanism of binding of human macrophage galactose-type lectin. Chemistry. (2019) 25:13945–55. 10.1002/chem.201902780 31404475

[12] Saeland E van Vliet SJ Backstrom M van den Berg VC Geijtenbeek TB Meijer GA. The C-type lectin MGL expressed by dendritic cells detects glycan changes on MUC1 in colon carcinoma. Cancer Immunol Immunother. (2007) 56:1225–36. 10.1007/s00262-006-0274-z 17195076 PMC11031027

[13] Higashi N Fujioka K Denda-Nagai K Hashimoto S Nagai S Sato T. The macrophage C-type lectin specific for galactose/N-acetylgalactosamine is an endocytic receptor expressed on monocyte-derived immature dendritic cells. J Biol Chem. (2002) 277:20686–93. 10.1074/jbc.M202104200 11919201

[14] van Vliet SJ van Liempt E Geijtenbeek TB van Kooyk Y. Differential regulation of C-type lectin expression on tolerogenic dendritic cell subsets. Immunobiology. (2006) 211:577–85. 10.1016/j.imbio.2006.05.022 16920496

[15] van Vliet SJ Gringhuis SI Geijtenbeek TB van Kooyk Y. Regulation of effector T cells by antigen-presenting cells via interaction of the C-type lectin MGL with CD45. Nat Immunol. (2006) 7:1200–8. 10.1038/ni1390 16998493

[16] van Vliet SJ Bay S Vuist IM Kalay H Garcia-Vallejo JJ Leclerc C. MGL signaling augments TLR2-mediated responses for enhanced IL-10 and TNF-alpha secretion. J Leukoc Biol. (2013) 94:315–23. 10.1189/jlb.1012520 23744646

[17] Li D Romain G Flamar AL Duluc D Dullaers M Li XH. Targeting self- and foreign antigens to dendritic cells via DC-ASGPR generates IL-10-producing suppressive CD4+ T cells. J Exp Med. (2012) 209:109–21. 10.1084/jem.20110399 22213806 PMC3260876

[18] Gu C Wang L Zurawski S Oh S. Signaling cascade through DC-ASGPR induces transcriptionally active CREB for IL-10 induction and immune regulation. J Immunol. (2019) 203:389–99. 10.4049/jimmunol.1900289 31175164

[19] Marcelo F Supekar N Corzana F van der Horst JC Vuist IM Live D. Identification of a secondary binding site in human macrophage galactose-type lectin by microarray studies: implications for the molecular recognition of its ligands. J Biol Chem. (2019) 294:1300–11. 10.1074/jbc.RA118.004957 30504228 PMC6349122

[20] Napoletano C Zizzari IG Rughetti A Rahimi H Irimura T Clausen H. Targeting of macrophage galactose-type C-type lectin (MGL) induces DC signaling and activation. Eur J Immunol. (2012) 42:936–45. 10.1002/eji.201142086 22531918

[21] Jantsch J Chakravortty D Turza N Prechtel AT Buchholz B Gerlach RG. Hypoxia and hypoxia-inducible factor-1 alpha modulate lipopolysaccharide-induced dendritic cell activation and function. J Immunol. (2008) 180:4697–705. 10.4049/jimmunol.180.7.4697 18354193

[22] Krawczyk CM Holowka T Sun J Blagih J Amiel E DeBerardinis RJ. Toll-like receptor-induced changes in glycolytic metabolism regulate dendritic cell activation. Blood. (2010) 115:4742–9. 10.1182/blood-2009-10-249540 20351312 PMC2890190

[23] Everts B Amiel E Huang SC Smith AM Chang CH Lam WY. TLR-driven early glycolytic reprogramming via the kinases TBK1-IKKvarepsilon supports the anabolic demands of dendritic cell activation. Nat Immunol. (2014) 15:323–32. 10.1038/ni.2833 24562310 PMC4358322

[24] Pearce EJ Everts B. Dendritic cell metabolism. Nat Rev Immunol. (2015) 15:18–29. 10.1038/nri3771 25534620 PMC4495583

[25] Dominguez-Andres J Arts RJW Ter Horst R Gresnigt MS Smeekens SP Ratter JM. Rewiring monocyte glucose metabolism via C-type lectin signaling protects against disseminated candidiasis. PLoS Pathog. (2017) 13:e1006632. 10.1371/journal.ppat.1006632 28922415 PMC5619837

[26] Campbell NK Fitzgerald HK Fletcher JM Dunne A. Plant-derived polyphenols modulate human dendritic cell metabolism and immune function via AMPK-dependent induction of heme oxygenase-1. Front Immunol. (2019) 10:345. 10.3389/fimmu.2019.00345 30881359 PMC6405514

[27] Malinarich F Duan K Hamid RA Bijin A Lin WX Poidinger M. High mitochondrial respiration and glycolytic capacity represent a metabolic phenotype of human tolerogenic dendritic cells. J Immunol. (2015) 194:5174–86. 10.4049/jimmunol.1303316 25917094

[28] Rodriguez-Prados JC Traves PG Cuenca J Rico D Aragones J Martin-Sanz P. Substrate fate in activated macrophages: a comparison between innate, classic, and alternative activation. J Immunol. (2010) 185:605–14. 10.4049/jimmunol.0901698 20498354

[29] Jha AK Huang SC Sergushichev A Lampropoulou V Ivanova Y Loginicheva E. Network integration of parallel metabolic and transcriptional data reveals metabolic modules that regulate macrophage polarization. Immunity. (2015) 42:419–30. 10.1016/j.immuni.2015.02.005 25786174

[30] Geeraerts X Bolli E Fendt SM Van Ginderachter JA. Macrophage metabolism as therapeutic target for cancer, atherosclerosis, and obesity. Front Immunol. (2017) 8:289. 10.3389/fimmu.2017.00289 28360914 PMC5350105

[31] Joshi NA Fass JN Sickle: A Sliding-Window, Adaptive, Quality-Based Trimming Tool for FastQ files (Version 1.33) [Software]. (2011). Available online at: https://github.com/najoshi/sickle

[32] Andrews S FastQC: A Quality Control Tool for High Throughput Sequence Data. (2010). Available online at: http://www.bioinformatics.babraham.ac.uk/projects/fastqc

[33] Yates A Akanni W Amode MR Barrell D Billis K Carvalho-Silva D. Ensembl 2016. Nucleic Acids Res. (2016) 44:D710–6. 10.1093/nar/gkv1157 26687719 PMC4702834

[34] Li H Handsaker B Wysoker A Fennell T Ruan J Homer N. The sequence alignment/map format and SAMtools. Bioinformatics. (2009) 25:2078–9. 10.1093/bioinformatics/btp352 19505943 PMC2723002

[35] Core Team R R: A Language and Environment for Statistical Computing. Vienna: R Foundation for Statistical Computing (2014). Available online at: http://www.R-project.org/

[36] Liao Y Smyth GK Shi W. featureCounts: an efficient general purpose program for assigning sequence reads to genomic features. Bioinformatics. (2014) 30:923–30. 10.1093/bioinformatics/btt656 24227677

[37] Robinson MD McCarthy DJ Smyth GK. edgeR: a Bioconductor package for differential expression analysis of digital gene expression data. Bioinformatics. (2010) 26:139–40. 10.1093/bioinformatics/btp616 19910308 PMC2796818

[38] McCarthy DJ Chen Y Smyth GK. Differential expression analysis of multifactor RNA-Seq experiments with respect to biological variation. Nucleic Acids Res. (2012) 40:4288–97. 10.1093/nar/gks042 22287627 PMC3378882

[39] Shannon P Markiel A Ozier O Baliga NS Wang JT Ramage D. Cytoscape: a software environment for integrated models of biomolecular interaction networks. Genome Res. (2003) 13:2498–504. 10.1101/gr.1239303 14597658 PMC403769

[40] Bindea G Mlecnik B Hackl H Charoentong P Tosolini M Kirilovsky A. ClueGO: a Cytoscape plug-in to decipher functionally grouped gene ontology and pathway annotation networks. Bioinformatics. (2009) 25:1091–3. 10.1093/bioinformatics/btp101 19237447 PMC2666812

[41] Luo W Brouwer C. Pathview: an R/Bioconductor package for pathway-based data integration and visualization. Bioinformatics. (2013) 29:1830–1. 10.1093/bioinformatics/btt285 23740750 PMC3702256

[42] Luo W Pant G Bhavnasi YK Blanchard SG Jr Brouwer C. Pathview Web: user friendly pathway visualization and data integration. Nucleic Acids Res. (2017) 45:W501–8. 10.1093/nar/gkx372 28482075 PMC5570256

[43] Wu AM Sugii S Coding and classification of D-galactose, N-acetyl-D-galactosamine, and beta-D-galp-[1-]3(4)]-beta-D-glcpnac, specificities of applied lectins. Carbohydrate Res. (1991) 213:127–43. 10.1016/S0008-6215(00)90604-9

[44] van Vliet SJ Paessens LC Broks-van den Berg VC Geijtenbeek TB van Kooyk Y. The C-type lectin macrophage galactose-type lectin impedes migration of immature APCs. J Immunol. (2008) 181:3148–55. 10.4049/jimmunol.181.5.3148 18713985

[45] Giovanelli P Sandoval TA Cubillos-Ruiz JR. Dendritic cell metabolism and function in tumors. Trends Immunol. (2019) 40:699–718. 10.1016/j.it.2019.06.004 31301952

[46] Goda N Kanai M. Hypoxia-inducible factors and their roles in energy metabolism. Int J Hematol. (2012) 95:457–63. 10.1007/s12185-012-1069-y 22535382

[47] Al Tameemi W Dale TP Al-Jumaily RMK Forsyth NR. Hypoxia-modified cancer cell metabolism. Front Cell Dev Biol. (2019) 7:4. 10.3389/fcell.2019.00004 30761299 PMC6362613

