# Supplementary material for: Activation of the C-Type Lectin MGL by Terminal GalNAc Ligands Reduces the Glycolytic Activity of Human Dendritic Cells
Source: Front Immunol. 2020 Feb 25;11:305. doi: 10.3389/fimmu.2020.00305 (PMC7053379; doi:10.3389/fimmu.2020.00305)
Supplement: Supplementary file 2 [file Data_Sheet_1.pdf]

Supplementary Material

1 Supplementary Figures and Tables

1.1 Supplementary Figures

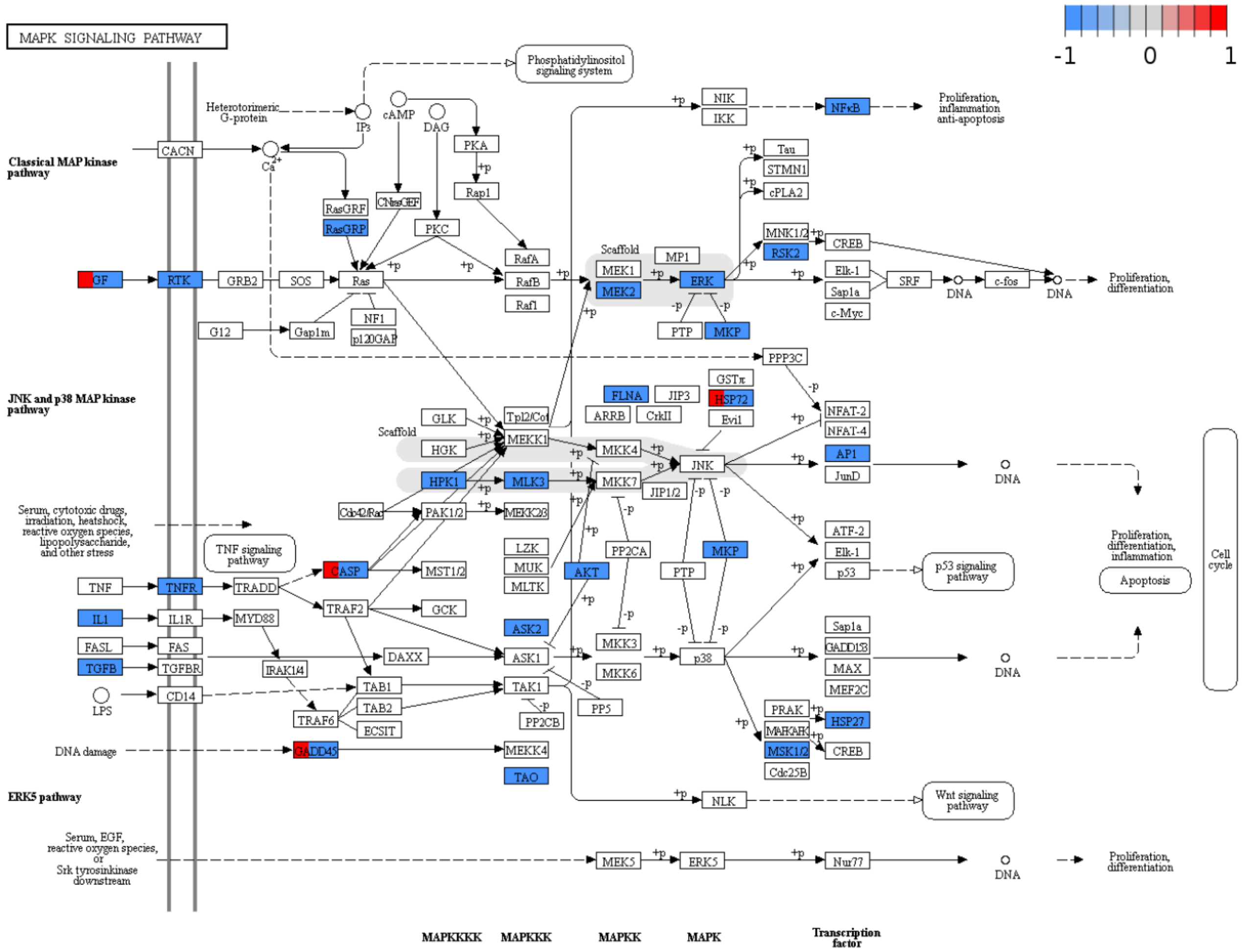

Figure S1. GalNAcβ1-4Gal alters the expression of genes involved in the MAPK signaling pathway. The 1106 differentially expressed genes after overnight stimulation of moDCs with GalNAcβ1-4Gal glycodendrimers were subjected to pathway analysis and visualization in the MAPK signaling pathway.

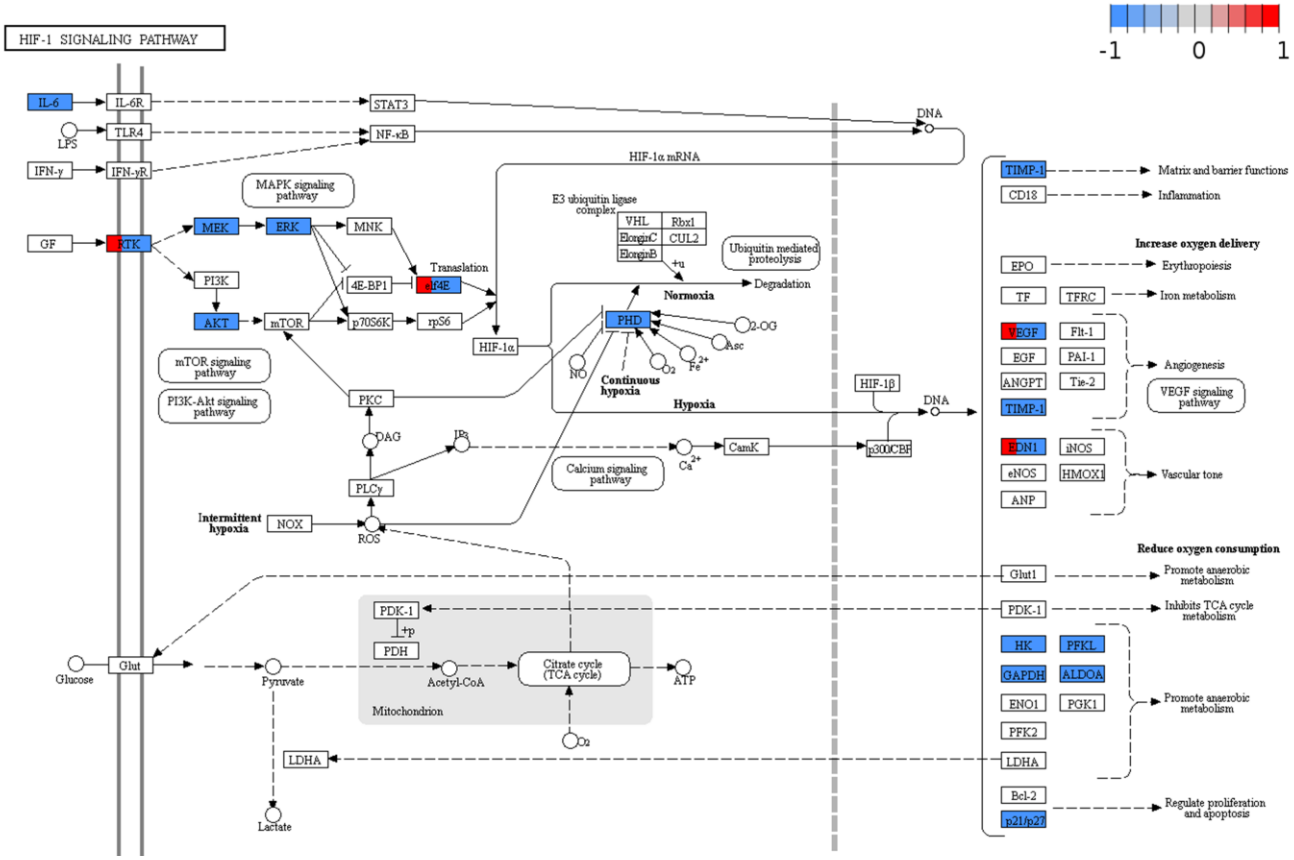

Figure S2. GalNAcβ1-4Gal reduces the expression of genes involved in the HIF-1 signaling pathway. The 1106 differentially expressed genes after overnight stimulation of moDCs with GalNAcβ1-4Gal glycodendrimers were subjected to pathway analysis and visualization in the HIF-1 signaling pathway.

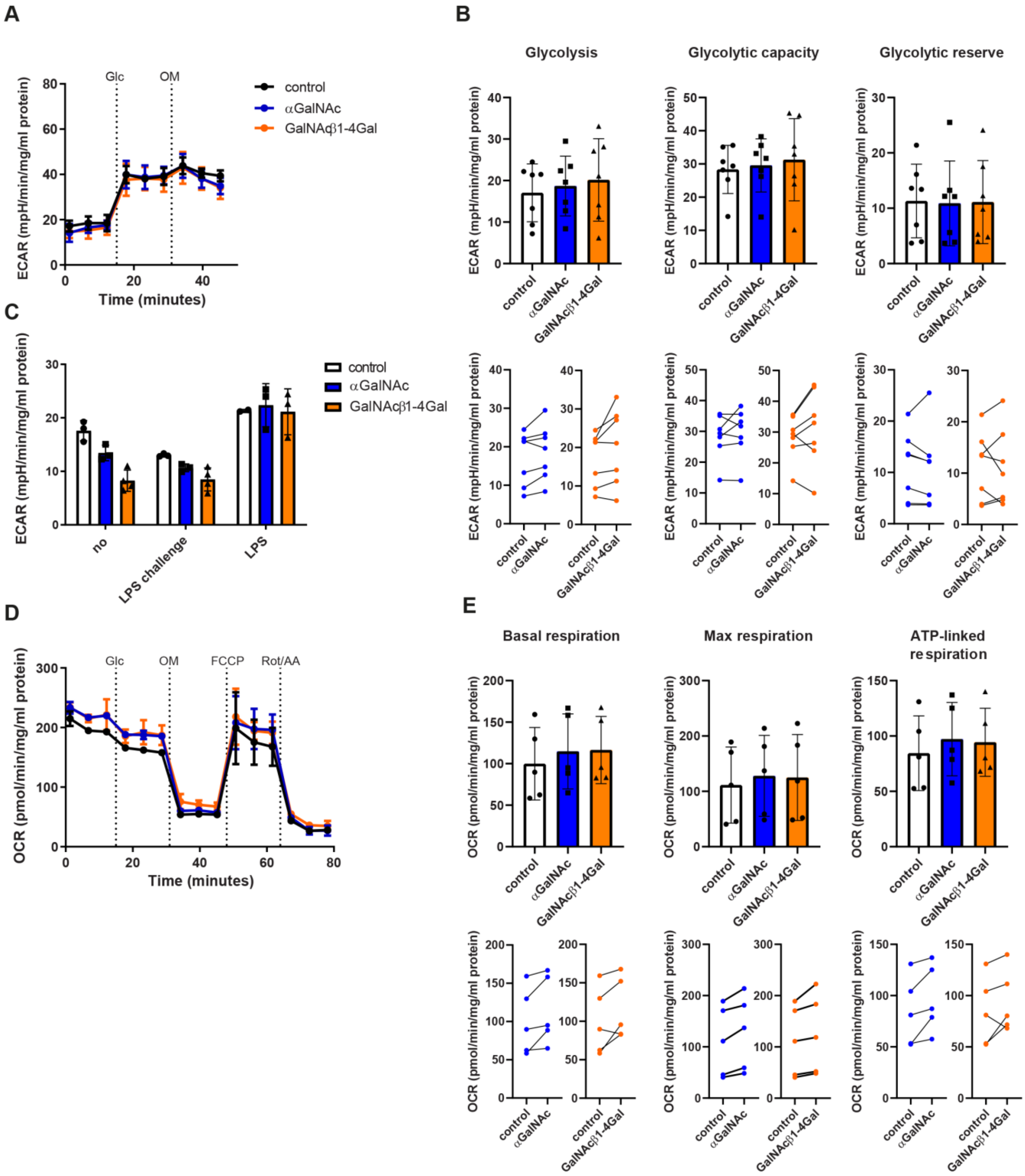

Figure S3. Reduced glycolytic activity in glycan-treated moDCs is overruled by strong TLR stimulation. (A-B, D-E) moDCs were stimulated overnight with αGalNAc (blue), GalNAcβ1-4Gal (orange) or control (black) glycodendrimers in the presence of LPS and subsequently subjected to extracellular flux analysis. Data was normalized based on protein content. **(A)** During extracellular flux analysis, ECAR in response to treatment with Glucose (Glc) and Oligomycin (OM) was measured. One representative of seven independent experiments is depicted. Error bars represent standard

deviation of 4-6 technical replicate measurements. **(B)** Glycolysis, glycolytic capacity, and glycolytic reserve were determined using the ECAR levels before and after treatment with Glc and OM (n=7). Individual donors are depicted in the line graphs (lower panels). **(C)** moDCs were stimulated overnight with αGalNAc (blue), GalNAcβ1-4Gal (orange) or control (black) glycodendrimers in the absence (medium, LPS challenge) or presence of LPS (LPS). To perform the LPS challenge, LPS was added 1 h prior to extracellular flux analysis to the overnight stimulation with the glycodendrimers. Data was normalized based on protein content and glycolysis was determined using the ECAR levels before and after Glc treatment. One representative of two independent experiments is depicted. Error bars represent standard deviation of 2-4 technical replicate measurements. **(D)** During extracellular flux analysis, OCR in response to treatment with Glc, OM, FCCP and rotenone plus antimycin A (Rot/AA) was measured. One representative of five independent experiments is depicted. Error bars represent standard deviation of 4-6 technical replicate measurements. **(E)** Basal respiration, maximal respiration and ATP-linked respiration were determined using the OCR levels before and after treatment with OM, FCCP and Rot/AA. Error bars represent of 5 independent experiments. Individual donors are depicted in the line graphs (lower panels).
